# *Theileria annulata*-transformed cell lines are efficient antigen-presenting cells for in vitro analysis of CD8 T cell responses to bovine herpesvirus-1

**DOI:** 10.1186/1297-9716-42-119

**Published:** 2011-12-19

**Authors:** Jane Hart, Niall D MacHugh, W Ivan Morrison

**Affiliations:** 1 The Roslin Institute and Royal (Dick) School of Veterinary Studies, University of Edinburgh, Easter Bush, Midlothian EH25 9RG, Scotland, UK

## Abstract

Continuously growing cell lines infected with the protozoan parasite *Theileria annulata *can readily be established by in vitro infection of leukocytes with the sporozoite stage of the parasite. The aim of the current study was to determine whether such transformed cell lines could be used as antigen presenting cells to analyse the antigenic specificity of bovine CD8 T cell responses to viral infections. Bovine herpes virus 1 (BHV-1), which is known to induce CD8 T cell responses, was used as a model. *T. annulata*- transformed cells were shown to express high levels of CD40 and CD80 and were susceptible to infection with BHV-1, vaccinia and canarypox viruses. The capacity of the cells to generate antigen-specific CD8 T cell lines was initially validated using a recombinant canarypox virus expressing a defined immunodominant *T. parva *antigen (Tp1). Autologous *T. annulata*-transformed cells infected with BHV-1 were then used successfully to generate specific CD8 T cell lines and clones from memory T cell populations of BHV-1-immune animals. These lines were BHV-1-specific and class I MHC-restricted. In contrast to previous studies, which reported recognition of the glycoproteins gB and gD, the CD8 T cell lines generated in this study did not recognise these glycoproteins. Given the ease with which *T. annulata*-transformed cell lines can be established and maintained in vitro and their susceptibility to infection with poxvirus vectors, these cell lines offer a convenient and efficient in vitro system to analyse the fine specificity of virus-specific CD8 T cell responses in cattle.

## Introduction

CD8^+ ^T cells are important mediators of immunity to many viral infections [1]. They recognise antigenic peptides presented in association with MHC class I molecules on the surface of infected cells and respond by lysing the cells or secreting cytokines that can limit viral replication and mediate recruitment of inflammatory cells. Although CD8 T cell responses have been documented for a number of viral infections in cattle, including bovine herpes virus 1 (BHV-1) [2-6], there have been few analyses of the fine antigenic specificity of these responses. BHV-1 is an important cause of respiratory disease in cattle [7] and, as with other related alpha herpesviruses, CD8 T cell responses are considered to have a key role in controlling persistent infection.

Many of the studies of CD8 T cell responses in cattle have relied on analyses of ex vivo T cell populations following a primary in vitro antigenic stimulation. Given the relatively low frequencies of virus-specific CD8 T cells in circulating memory T cell populations (typically ranging from 1/500 to 1/20 000 in human peripheral blood) [8,9], such assays have limited sensitivity. The establishment in vitro of cell lines enriched for virus-specific T cells provides a more sensitive means of dissecting the specificity of the response. Such systems can also permit the generation of T cell clones, allowing analysis of responses at the clonal level. Efficient activation of specific CD8 T cells requires, in addition to antigenic recognition, interaction with co-stimulatory molecules such as CD80 and CD86 on the antigen presenting cells (APC) [10]. Since expression of these proteins is generally confined to professional APC, dendritic cells and monocytes have frequently been used as APC for stimulation of CD8 T cell cultures [11,12]. In humans, Epstein-Barr virus (EBV) transformed B cell lines have proved to be efficient stimulators of CD8 T cells in vitro and have been used extensively to study responses to a variety of viruses [13-16], in most cases employing poxvirus vectors expressing individual viral proteins [16,17].

The protozoan parasites *Theileria parva *and *T. annulata *infect bovine mononuclear leukocytes and induce them to proliferate [18]. By associating with the mitotic apparatus of the host cells, the parasite is able to divide at the same time as the cell ensuring that infection is maintained in the daughter cells. Infection of mononuclear leukocytes in vitro transforms them into continuously dividing cells, which can be maintained indefinitely in culture. These cell lines express surface MHC class I and II [19]. They have been used extensively in studies of CD8 T cell responses to the respective Theileria parasites, and have facilitated the generation of parasite-specific CD8 T cell lines and clones for analysing the antigenic specificity of the responses [20-22].

In view of the immunostimulatory properties of Theileria-infected cells, the current study set out to investigate whether *T. annulata-*transformed cells could be used as APC to analyse bovine responses to viral pathogens, focusing initially on BHV-1 as a model.

## Materials and methods

### Animals

Friesian/Holstein cattle aged approximately 18 months at the outset of the experiments were used in the study. Their MHC class I types were determined using a combination of serological typing with monoclonal antibodies [23] and allele-specific PCR [24]. Animals used for analysis of responses to BHV-1 were vaccinated with a live attenuated BHV-1 vaccine (Rispoval IBR marker live, Pfizer Animal Health, Walton Oaks, Surrey, U.K.) and challenged intranasally with 2 × 10^7 ^TCID_50 _BHV-1 Iowa strain (kindly provided by Moredun Scientific, Roslin, UK) 4 weeks later. Responses of these animals were studied 9-14 months post challenge. An animal homozygous for the MHC class I A18 haplotype was used as a donor of immune *T. parva*-specific T cells; this animal had been immunised with *T. parva *(Muguga strain) by infection with tick-derived sporozoites and simultaneous treatment with long-acting oxytetracycline as described previously [25].

### Viruses

BHV-1 Cooper strain was provided by Dr Kim Willoughby, Moredun Research Institute. Stocks of BHV-1 were obtained by infection of bovine turbinate (BT) cell lines at multiplicity of infection of 0.01 in Iscoves modified Dulbecco's medium containing 10% heat-inactivated foetal calf serum (FCS), penicillin/streptomycin, L-glutamine 0.3 mg/mL (Invitrogen, Paisley, UK) and 50 μM 2-mercaptoethanol. Cell-associated virus was released by freezing and thawing of the cells and the titres of the resultant virus suspensions were determined using an infectivity assay on BT cells and expressed as 50% tissue culture infective dose (TCID_50_)/mL. All cells were incubated in a humidified incubator at 37°C in the presence of 5% CO_2_.

Recombinant canarypox viruses expressing the *T. parva *antigens Tp1 and Tp2 (Tp1-canarypox and Tp2-canarypox), and attenuated NYVAC vaccinia viruses expressing the BHV-1 glycoproteins gD, gC or gB (NYVAC-gD, gC, gB) were provided by Dr Jean-Christophe Audonnet, Merial, Lyon, France.

### Generation of Theileria transformed cell lines

Peripheral blood mononuclear cells (PBMC) were isolated from venous blood by density gradient centrifugation using Ficoll-Paque as described [26]. Theileria-transformed cell lines were established and maintained in RPMI 1640 culture medium (Invitrogen) containing 10% heat-inactivated FCS, penicillin/streptomycin 100 U/mL and 0.3 mg/mL L-glutamine (RPMI complete medium). PBMC were infected in vitro with sporozoites of the *T. annulata *Ankara isolate (TaA) or the *T. parva *Muguga isolate from suspensions of parasites cryopreserved at approximately 2 tick equivalents per mL in 7.5% glycerol, as previously described [27]. Briefly, the sporozoites were thawed rapidly and subjected to three 2-fold dilutions in RPMI complete medium at 10 min intervals. Aliquots of 1 mL were added to 1 mL of PBMC at 4 × 10^6^/mL in the wells of 24-well plates and incubated at 37°C in 10% CO_2_. Approximately half of the medium was removed and replaced with fresh medium every 3-4 days. Outgrowth of transformed cells was first detected after 10-20 days and fully transformed cell lines became established by 3-4 weeks. Such lines were maintained by passage every 2-4 days. They grew to a density of 1-2 × 10^6^/mL and had population doubling times of 18-24 h.

### Flow cytometry

Populations of T cells were analysed by single-colour indirect immunoflourescence staining and flow cytometry, using monoclonal antibodies specific for CD3 (MM1A -IgG1), CD4 (IL-A12 - IgG2a), CD8 (IL-A51 - IgG1), the γδ T-cell receptor (GB21A - IgG2b) and the γδ T-cell-specific marker WC1 (CC15 - IgG2a), followed by a fluorochrome-labelled anti-mouse Ig secondary antibody (Sigma-Aldrich, Dorset, UK). Additional monoclonal antibodies specific for IgM (IL-A30 - IgG1), sirp1α (IL-A24 -IgG1), class I MHC (IL-A88- IgG2a), class II DR (IL-A21 - IgG2a), class II DQ (CC158 - IgG2a), CD40 (IL-A156 -IgG1), CD80 (IL-A159 - IgG1) and CD86 (IL-A190 - IgG1) were used to examine the phenotype of *T. annulata*-transformed cells. Infection of *T. annulata*-transformed cells with BHV-1 or recombinant canarypox viruses expressing BHV-1 glycoproteins was also assessed by indirect immunofluorescence staining using monoclonal antibodies specific for the BHV-1 glycoprotein D or gC [28], kindly provided by Dr G. Letchworth, USDA Arthropod-Borne Animal Diseases Research Lab, Laramie, Wyoming, USA), followed by FITC-conjugated anti-mouse Ig. Stained cells were analysed using a FACScalibur flow cytometer (Becton-Dickinson, Mountain View, CA, USA).

In order to assess T cell proliferation following antigenic stimulation, cells incubated with carboxy-fluorescein succinyl ester (CFSE) prior to culture [29] were harvested after stimulation and stained for T cell subset markers. Briefly, PBMC were washed in PBS 0.1% BSA then resuspended in 10 μM CFSE in PBS containing 0.1% BSA at approximately 2 × 10^7^/mL and incubated for 10 min at 37°C. Staining was then stopped by the addition of 5 volumes of ice-cold cell culture medium containing 10% FCS and the cells incubated on ice for 10 min. The cells were then washed 3 times in ice-cold culture medium before adding to APC for antigenic stimulation (see below). Cells harvested after 7 days were stained by indirect immunofluorescence with monoclonal antibodies specific for CD4, CD8 and γδ T cells, followed by a phycoerythrin-labelled secondary anti-mouse Ig (Sigma, UK) and analysed by 2-colour flow cytometry.

### Infection of cells with virus for immunological assays

Theileria-infected cells were infected with BHV-1 (Cooper strain) or recombinant pox viruses at a multiplicity of infection of 2 and incubated for 1 h, before washing 3 times to remove free virus from the supernatant. The infected cells were then incubated overnight before using as antigen-presenting cells or as targets for in vitro cytotoxicity assays. The levels of infection of the cells with BHV-1 were monitored by indirect immunofluorescence staining of the cells with the gD-specific antibody. Infected cells used as stimulators in in vitro cultures were irradiated by exposure to 50 Gy of gamma irradiation from a ^137^Cs source.

### Generation of antigen specific T cell lines

Antigen-specific T cell lines were generated from immune animals using a protocol similar to that described previously for generating Theileria-specific T cell lines [26]. Aliquots of 4 × 10^6 ^PBMC and 2 × 10^5 ^gamma-irradiated APC in 2 mL of RPMI complete medium were added to the wells of 24-well plates and cultured for 7 days. Viable cells were harvested and stimulated again by addition of fresh APC at the same ratios. After a further 7 days, the cell cultures were enriched for CD8 T cells by depletion of γδ T cells and CD4 T cells by complement-mediated lysis, using the monoclonal antibodies IL-A12 and CC15 specific for CD4 and WC1 respectively, as described [30]. Normal rabbit serum at a dilution of 1:3 was used as a source of complement. The remaining cells were incubated overnight and the level of depletion determined by immunofluorescence staining and flow cytometry. The CD8-enriched populations were then subjected to a further stimulation with APC at a ratio of 10:1 APC to T cells with addition of 100 U/mL recombinant human IL-2 (Chiron, Emryville, CA, USA). After incubation for 7 days, the cells were cloned at limiting dilutions in 96-well round-bottomed plates by seeding at 0.3, 1 and 3 cells per well with 2 × 10^4 ^irradiated autologous PBMC per well as filler cells, 5 × 10^3 ^irradiated APC per well and 100 U/mL IL-2. Two weeks later, cells from wells showing good growth at clonal frequencies were transferred into 48-well plates and expanded by restimulating with 2.5 × 10^5 ^per well autologous irradiated APC and 100 U/mL recombinant human IL-2. The phenotype of the clones was determined by mAb staining and flow cytometry.

### Interferon gamma assay

Interferon gamma (IFN-γ) in supernatants was measured using a sandwich ELISA in Immulon 4HBX flat-bottomed microtitre ELISA plates (Thermo Fisher scientific, Roskilde, Denmark), using non-competing monoclonal antibodies specific for bovine IFN-γ (Clones CC330 and CC302, AbD Serotec, Kidlington, UK). The latter was conjugated with biotin and binding detected with horseradish peroxidase-conjugated streptavidin (AbD Serotec) followed by addition of TMB (3,3',5,5'-tetramethylbenzidine) substrate solution (BD Biosciences, Oxford, UK). A standard curve for IFN-γ determination was generated using a series of doubling dilutions of bovine IFN-γ (AdD Serotec). The IFN-γ concentration was then calculated by correlation to the standard curve generated by the IFN-γ standards.

### Cytotoxicity assays

A 4-hour ^111^indium release assay was used to assess the cytotoxicity of expanded CD8 T cell clones as previously described [26]. Briefly, for screening of clones, 100 μL of cells were removed from each well of the 48-well plates and incubated with 5 × 10^3 111^In-labelled target cells in V-bottom 96-well plates. In subsequent assays, defined numbers of effector cells were used at a range of effector to target ratios. Plates were incubated at 37°C for 4 h in a humidified atmosphere of 5% CO_2 _in air. Control wells containing target cells alone to measure spontaneous release of indium or target cells with 0.2% Tween to measure maximal release of indium were included in all assays. Radioactivity in supernatants was measured using a gamma-counter and percent cytotoxicity was calculated as [(average cpm released - average spontaneous release cpm)/average total cpm released - average spontaneous cpm)] × 100.

## Results

### Phenotype of *T. annulata*-transformed cell lines

**Figure 1:**
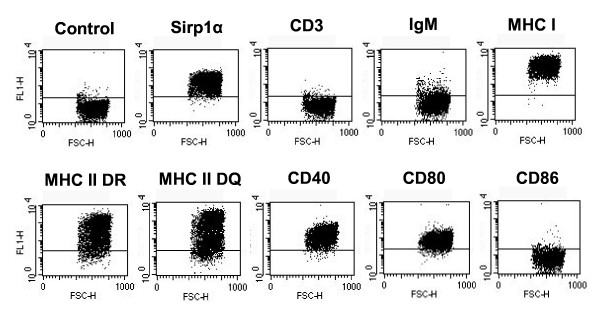
**Phenotype of a *T. annulata*-infected cell line (3430Ta) determined by indirect immunofluorescence staining and flow cytometry**.

*Theileria annulata*-transformed cell lines derived from 3 cattle exhibited virtually identical phenotypes when stained with a panel of monoclonal antibodies specific for bovine cell surface markers. The phenotype of one of the lines is illustrated in Figure 1. The cells were positive for the myeloid lineage marker sirp1α, but negative for CD3 and IgM; they expressed high levels of class I and both DR and DQ class II MHC, as well as CD40 and CD80, but did not express CD86.

### Susceptibility of *T. annulata*-transformed cells to infection with BHV-1 and poxvirus vectors

**Figure 2:**
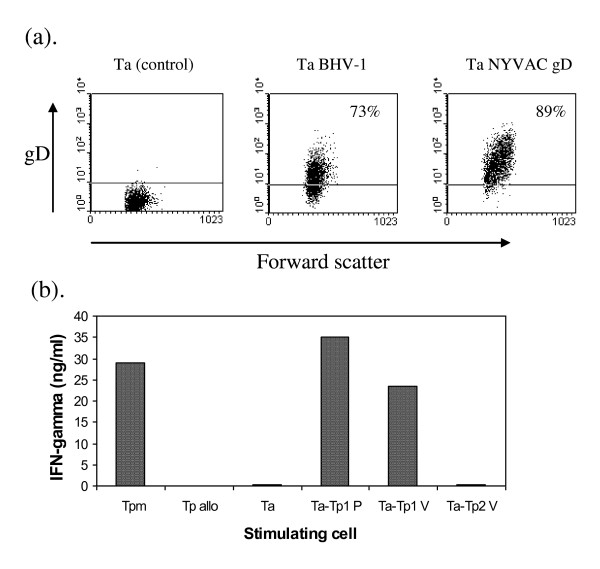
**Infection of *T. annulata*-transformed cells with BHV-1, recombinant vaccinia virus NYVAC-gD, and recombinant Tp1-canarypox virus**. (a). *T. annulata *transformed cells from animal 03430 were harvested 18 h after infection with BHV-1 or with NYVAC-gD and stained by indirect immunofluorescence with a monoclonal antibody specific for the BHV-1 protein gD. Control uninfected cells stained with the same antibody are shown in the left panel. Infected cells stained only with secondary antibody were also negative (data not shown). The percentages of positive cells are indicated within the panels. (b). Cells infected with Tp1-canarypox were tested for recognition by a CD8 T cell line specific for Tp1 from a *T. parva-*immune, class I MHC A18-homozygous cow, using an IFN-γ release assay. Results are shown for T cells stimulated with autologous or allogeneic *T. parva *transformed cells (Tp and Tp allo respectively), or autologous *T. annulata*-transformed cells pulsed with Tp1 _214-224 _peptide (Ta-Tp1 P), or infected with a canarypox virus expressing Tp1 (Ta-Tp1V), canarypox virus expressing Tp2 (Ta-Tp2 V) or uninfected (TA). Supernatants of T cell cultures collected 20 h after antigenic stimulation were assayed for IFN-γ using a specific ELISA. The results represent the mean of measurements from duplicate cultures. The experiment was repeated once with similar results.

The same 3 transformed cell lines were examined for their ability to support infection with BHV-1, and with a recombinant vaccinia virus (NYVAC strain) expressing the BHV-1 gD glycoprotein (NYVAC-gD). Cultures infected with 2 TCID_50_/cell of BHV-1 or 2 cfu/cell of NYVAC-gD typically contained 70-90% infected cells 18 h later, as indicated by specific surface staining with an anti-gD mAb (Figure 2a).

No suitable antibodies were available to test for infection with canarypox. Instead, *T. annulata*-transformed cells infected with a canarypox recombinant expressing the Tp1 *T. parva *antigen, were tested for recognition by a CD8 T cell line specific for Tp1 using an IFN-γ ELISA. The canarypox-infected, autologous *T. annulata *transformed cells resulted in IFN-γ secretion at levels similar to that obtained with *T. parva *transformed stimulators and *T. annulata *transformed cells pulsed with the Tp1 epitope peptide. Stimulation with the uninfected *T. annulata *transformed cells or cells infected with a control Tp2-canarypox resulted in only low levels of IFN-γ secretion (Figure 2b).

### Generation of a Tp1-specific CD8 T cell line using *T. annulata*-transformed cells

In cattle carrying the A18 class I MHC haplotype, the CD8 T cell response to *T. parva *is dominated by an epitope in the Tp1 antigen (Tp1_214-224_) [21,22]. This antigen was therefore used as a positive control in initial experiments to test the capacity of the *T. annulata *transformed cells to act as APC for in vitro induction of specific CD8 T cell responses. *T. annulata *transformed cells from an A18^+ ^*T. parva*-immune animal were infected with a recombinant canarypox virus expressing the Tp1 gene (Tp1 canarypox) and used as stimulator cells in cultures with autologous PBMC. Since previous studies have demonstrated that Theileria-transformed cells stimulate non-specific lymphocyte proliferation in PBMC from naïve animals, cultures stimulated with *T. annulata*-transformed cells with or without added Tp1 canarypox were compared for evidence of antigen-specific T cell proliferation by analysing loss of CFSE staining 7 days after the first ex-vivo stimulation.

**Figure 3:**
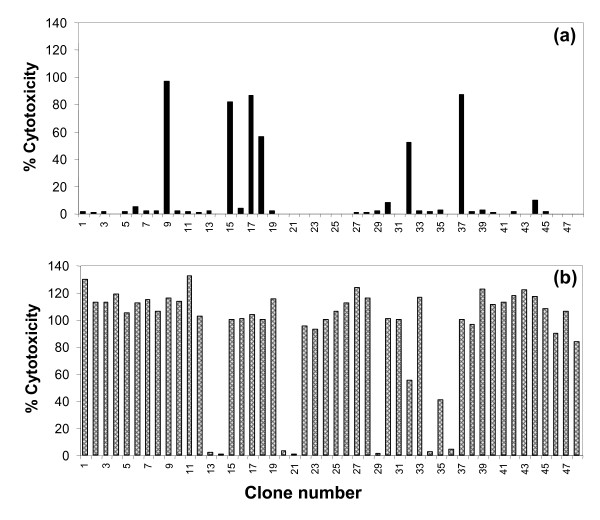
**Specificity of CD8 T cell clones generated from PBMC of a *T. parva*-immune animal by in vitro stimulation with autologous *T. annulata*-transformed cells infected with Tp1 canarypox**. Forty-eight CD8 T cell clones were analysed for cytotoxicity in a 4-hour ^111^Indium release assay using as targets autologous *T. annulata *tranformed cells alone (a) or pulsed with a peptide representing a dominant epitope (Tp1_214-224_) in the Tp1 antigen (Tp1) (b). The majority of clones are specific for Tp1_214-225_.

**Figure 4:**
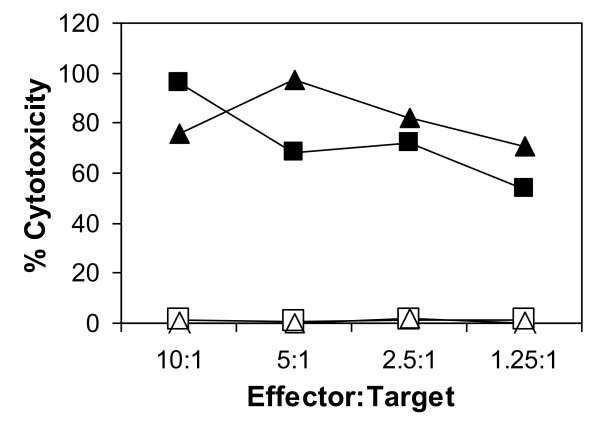
**CD8 T cell clones specific for the Tp1 antigen show MHC class I-restricted cytotoxicity**. Two Tp1 specific CD8 T cell clones were tested in a 4-hour ^111^Indium release cytotoxicity assay against autologous (solid black symbols) and MHC mis-matched (open symbols) Tp1_214-224_-pulsed targets at defined effector:target ratios.

Cultures stimulated with *T. annulata *transformed cells expressing Tp1 contained a higher percentage of proliferating CD4 T cells (33.7% cf 15.7%) and CD8 T cells (10.5% cf 1.6%) and a lower proportion of proliferating γδ T cells (11.8% cf 30.2%) compared to cultures stimulated with the *T. annulata *transformed cells alone (data not shown), indicating the presence of CD4 and CD8 T cells reactive with the recombinant virus. To determine whether the proliferating CD8 T cells were specific for Tp1, T cell clones were established from a CD8 enriched T cell population and screened for their ability to kill autologous target cells pulsed with a peptide representing the Tp1_214-224 _epitope. Of 48 T cell clones analysed, 41 clones lysed the peptide-pulsed target, giving levels of killing ranging from 41% to 100%; 6 of these clones also lysed unpulsed target cells (Figure 3). The remaining 35 cytotoxic clones did not kill either the unpulsed autologous target cell or an MHC-mismatched target cell pulsed with Tp1_214-224_. Representative data for 2 clones tested at a range of defined effector:target ratios are shown in Figure 4. Both clones showed highly specific killing at levels > 50% at the lowest effector to target ratio tested (1.25:1).

The results of these experiments indicate that *T. annulata*-transformed cells are effective as APC to present virus-expressed antigens for in vitro stimulation of CD8 T cells from immune animals.

### Generation of BHV-1 specific CD8 T cell lines

**Figure 5:**
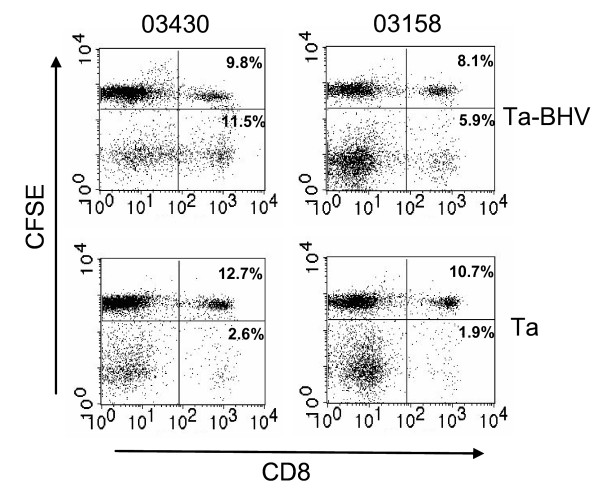
**Proliferative responses of CD8 T cells from BHV-1 immune animals to autologous *T. annulata*-transformed cells infected with BHV-1**. PBMC from two immune animals (03430 and 03158) were stimulated with autologous *T. annulata *transformed cells (Ta) or the same cells infected with BHV-1 (Ta BHV). Proliferation of CD8 T cells was examined by pre-incubation of the PBMC with CFSE and analysis of the cells after 7 days by two-colour immunofluorescence to detect CSFE and CD8. The percentages of CD8^+ ^cells in the cultures showing loss of CSFE staining (indicating cell division) and those retaining CFSE staining are shown in the lower right and upper right quadrants respectively.

In order to determine whether similar specific CD8 T cell lines could be obtained from BHV-1-immune animals, PBMC from 3 cattle previously vaccinated and challenged with BHV-1 were stimulated in vitro with autologous *T. annulata*-transformed cells infected with the virus. Analysis of CSFE-stained cultures from 2 of these animals, 7 days after primary ex vivo stimulation, revealed an increase in the percentage of responding CD8 T cells (11.5% cf 2.6% for 03430 and 5.9% cf 1.9% for 03158) in cultures stimulated with virus-infected cells compared to those stimulated with uninfected *T. annulata*-transformed cells (Figure 5). This was associated with a decrease in the content of proliferating γδ T cells (33.0% cf 60.0% for 03430 and 19.7% cf 36.4% for 03158) in both cultures.

CD8 T cell clones derived from these 3 lines, following depletion of other T cell subsets, were screened for cytotoxic activity. The number of T cell clones obtained from each animal and the number showing virus-specific cytotoxic activity are shown in Table 1. Cultures from all 3 animals yielded BHV-1-specific CD8 T cell clones, although cells from animal 03158 showed a low cloning efficiency and yielded only 2 specific clones. Out of a total of 81 cloned lines screened, 62 gave detectable killing of autologous BHV-1-infected cells, ranging from 6% to 100%, but gave no killing of uninfected autologous cells or allogeneic cells infected with BHV-1. An additional 2 clones exhibited non-specific killing of all 3 target cells.

**Table 1:** Specificity of CD8 T cell clones obtained from 3 BHV-1 immune animals by in vitro stimulation with autologous *T.annulata*-transformed cells infected with BHV-1

Animal	Number of T cell clones^1^			Mean % lysis (range)
		
	Total	BHV-1-specific	MHC class I restricted	
03109	44	40	39	40 (6-100)
03430	29	22	21	15 (5-42)
03158	8	2	2	27 (15-38)

^1^The specificity of the clones was examined using and a 4-hour ^111^In release cytotoxicity assay.

**Figure 6:**
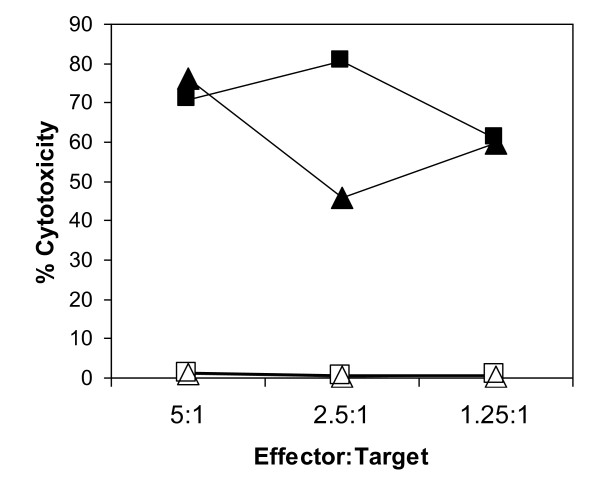
**BHV-1 specific CTL clones display MHC class I restricted cytotoxicity**. Two CD8 T cell clones were tested in a 4-hour ^111^Indium release cytotoxicity assay, at a range of effector:target ratios, using autologous (solid black symbols) and MHC mis-matched (open symbols) target cells.

The cytolytic activity of selected virus-specific clones was assayed on the appropriate target cells over a range of effector to target ratios. Representative data obtained with two clones derived from animal 3109, shown in Figure 6, demonstrate that the activity is highly specific for autologous BHV-1 infected targets.

The results demonstrate that autologous *T. annulata *transformed cells are highly effective as APC for stimulation of BHV-1 specific CD8 T cells from immune animals.

### The CD8 T cell response includes clones restricted by both MHC haplotypes

**Figure 7:**
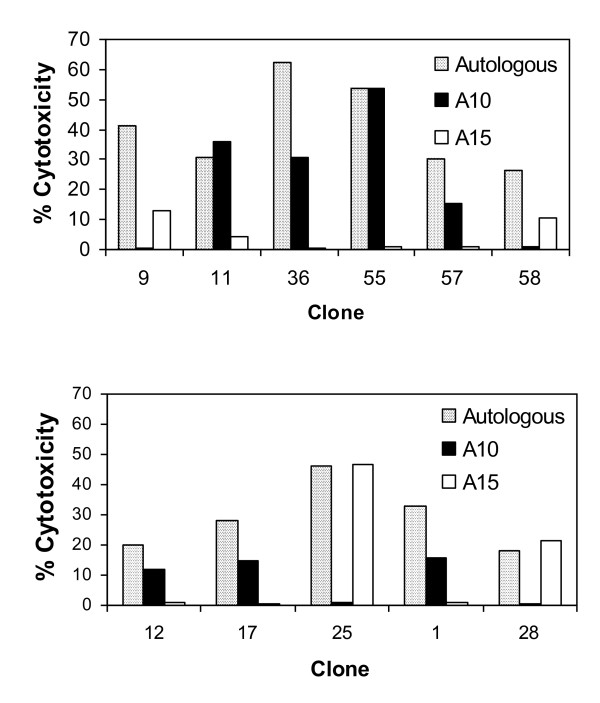
**MHC restriction specificities of BHV-1-specific CD8 T cell clones generated from two cattle expressing the A10 and A15 class I MHC haplotypes**. T cell clones from animals 03109 (top) and 03430 (bottom) were analysed in a 4-hour ^111^Indium release cytotoxicity assay using BHV-1 infected *T. annulata*-transformed target cells from the autologous animal (stipled bars) and from animals sharing the A10 haplotype (solid bars) or the A15 haplotype (open bar) with the effectors.

CD8 T cell clones that continued to show good growth following further passage were assayed on target cells matched for either of the MHC haplotypes (phenotypes A10/A10 and A15/A11) of the donor animal. Twenty CD8 T cell clones derived from two animals (03109 and 03430), both expressing the A10 and A15 class I haplotypes, were examined. The levels of killing obtained with 3 clones were too low to provide interpretable results. Of the remaining 17 clones, 10 clones (6 from 03109 and 4 from 03430) only killed targets expressing A10, while 5 clones (3 from 03109 and 2 from 03430) only killed targets expressing A15. Representative results obtained with 11 of these clones are shown in Figure 7. Two additional clones from animal 03430, although apparently virus-specific, killed targets expressing either A10 or A15, suggesting that either these were not true clones or they recognised viral products presented by a non-polymorphic MHC product. The results further confirm the class I MHC restriction of a majority of the CD8 T cell clones and demonstrate that in both animals the response is restricted by MHC products of both haplotypes indicating the presence of least two peptide-MHC specificities.

### The CD8 T cell lines do not recognise BHV glycoproteins gB, gC and gD

Since a previous study reported evidence that the CD8 T cell response to BHV-1 included T cells specific for the viral glycoproteins gB and gD, autologous target cells infected with recombinant vaccinia viruses expressing gB, gC or gD were tested for recognition by uncloned CD8 T cell lines from animals 03109 using an in vitro cytotoxicity assay. No lysis of any of these targets was observed (data not shown). Additional experiments with uncloned lines from 03109 and 03430 using an IFN-γ release assay also gave negative results, indicating that these viral glycoproteins are not dominant antigenic targets of these T cell lines.

## Discussion

The present study has demonstrated that virus-infected *T. annulata*-transformed cells can be used for in vitro generation and maintenance of virus-specific CD8 T cell lines from immune cattle. The parasitized cells were shown to be susceptible to infection with BHV-1, vaccinia and fowlpox viruses. Following validation of their capacity to stimulate specific CD8 T responses, using recombinant fowlpox virus expressing a defined *T. parva *antigen (Tp1), the system was used successfully to generate BHV-1-specific CD8 T cell lines and clones from BHV-1-immune animals. Although clearly virus-specific, these CD8 T cell lines did not recognise the glycoproteins gB, gC and gD, reported previously as targets of the CD8 T cell response.

*T. annulata *transformed cell lines have been used successfully to generate parasite-specific CD8 T cell lines from *T. annulata*-immune animals [31]. Parasitised cell lines can readily be established by infection of blood leukocytes in vitro with parasites from cryopreserved stocks and can be maintained indefinitely in culture and crypreserved with little loss of viability. In contrast to *T. parva*-transformed lines [32], *T. annulata*-infected cells do not produce IFN-γ or other T cell-specific cytokines that might confound assays of T cell responses [33]. In the present study, we sought to investigate whether these properties of *T. annulata*-transformed lines would enable them to be used as APC to investigate T cell responses to viral infections. Such an application would be analogous to the use in humans of EBV-transformed lines, which are infectable with recombinant poxviruses and have been used extensively to study the specificity of CD8 T cell responses to a number of viral infections including hepatitis C virus [16], HIV [34], and CMV [15]. They have been shown to be effective in stimulating a response when very low numbers of memory T cells are present.

Previous studies have shown that *T. annulata *can infect monocyte and B cells [35]. Phenotypic analysis of 3 cell lines used in the present study demonstrated that they express the myeloid cell marker sirp1α but not CD3 or surface IgM, suggesting that they were of monocyte origin. The results confirmed that the parasitised cells have high levels of both class I and class II MHC and also demonstrated that they express CD40 and CD80, both of which have been implicated as important co-stimulatory ligands for CD8 T cells [36,37]. The 3 cell lines were also shown to be susceptible to infection with recombinant poxviruses thus enabling presentation of defined antigens to potentially responsive T cell populations. A *T. parva *antigen, Tp1, was initially used to test for induction of CD8 T cell responses. This antigen was chosen because it is a highly dominant target in CD8 T cell responses of Friesian/Holstein cattle expressing the A18 class I MHC haplotype; over 70% of the *T. parva*-specific CD8 T cells from A18-homozygous animals immunised with live *T. parva *have been shown to be specific for an 11-mer epitope in Tp1 [21,22]. Importantly, Tp1-specific T cells do not cross-react with *T. annulata. T. annulata*-transformed cells infected with a recombinant canarypox virus expressing Tp1 were shown, at the outset of the study, to be recognised by a Tp1-specific CD8 T cell line, thus indicating adequate expression of Tp1. Canarypox viruses are replication deficient in mammalian cells and hence infection of the APC prior to addition of the responder T cells avoids possible infection of the responding cell populations. CFSE staining of cultures of PBMC from *T. parva *immune animals stimulated with the Tp1-canarypox-infected cells demonstrated specific proliferation of CD8 T cells, and clonal analysis of the CD8 T cell component revealed a high frequency of MHC class I restricted T cell clones specific for the dominant epitope in Tp1. These findings confirmed that *T. annulata *transformed cells were effective as APC for activation of antigen-specific CD8 T cells and that infection with recombinant canarypox was an effective means of delivery of antigen to the APC.

BHV-1 infects a number of cell types including blood monocytes and epithelial cells and causes disease affecting the respiratory and reproductive tracts. Following recovery from acute infection, the virus persists in a latent state in dorsal root ganglia [7]. In common with herpesvirus infections in other species, such as HSV-1 [38], MHC restricted cytolytic T cells have been identified in animals immune to BHV-1 [2,39] and are believed to have an important role in controlling infection with the virus and in prevention of reactivation of the virus from latency.

In the present study, we showed that BHV-1 efficiently infects *T. annulata*-transformed cells and that these cells can be used as APC to generate and maintain virus-specific CD8 T cell lines. Virus-specific, MHC class I restricted CD8 T cell lines and clones were generated from all 3 BHV-1 immune animals studied, although cells from one animal showed a low cloning efficiency. Virus specificity was based on detection of cytotoxic activity. Since studies of CD8 T cell responses in humans and mice have shown that clones specific for a given antigen/epitope are functionally heterogeneous [40], it is possible that some of the non-cytolytic clones were also specific for BHV-1. Analysis of MHC restriction of CD8 T cell clones from 2 of the animals, which had the same MHC genotypes, demonstrated responses restricted by both class I haplotypes (A10 and A15), indicating that at least two viral epitopes are recognised by these animals. This is in contrast to CD8 T responses of cattle to *T. parva *and *T. annulata*, which in some animals are almost completely restricted by the products of one MHC haplotype [20,22].

The viral glycoproteins gC and gD have been identified as targets for CD8 T cell responses to BHV-1 in cattle [3], and CD8 T cell responses have been induced by gB and gD DNA vaccines in mice [41], and by gB DNA vaccines in cattle [42]. However, there have been no systematic studies of the antigenic specificity of the responses to BHV-1 infection. In contrast to the previous findings, analysis of CD8 T cells from animals in the current study indicated that they do not recognise the gB, gC and gD viral glycoproteins. This discrepancy may relate to the time after initial infection when the responses were examined. Our analyses focused on memory T cells in PBMC harvested 9-14 months after infection, whereas previous studies examined animals in the first 3 months after infection [3] and 2 weeks post vaccination [42]. Moreover, some of the previously reported experiments [3] used as stimulator cells APC incubated with UV-inactivated virus. Although herpes simplex virus 1 subjected to doses of UV irradiation that result in 10-fold reduction in progeny virus has been shown to retain expression of non-structural proteins [43], levels of expression by the completely UV-inactivated BHV-1 virus used in these previous bovine studies may have been reduced, thus biasing the response towards structural proteins processed from the viral inoculum. Thus, although animals may be capable of generating CD8 T cell responses to these glycoproteins, these responses may not represent a dominant component of the long-term memory CD8 T cell population. Alternatively, since it is well established that MHC genotype can influence the antigenic specificity of CD8 T cell responses [44], it is possible that only animals of certain MHC genotypes give strong CD8 T cell responses to these glycoproteins. Further experiments are underway using the CD8 T cell lines generated by the current study to screen for the target BHV-1 antigens.

Since *T. annulata *is confined to sub-tropical regions where the tick vector species occur, use of the infected cells as APC outside these regions will not be confounded by specific T cell responses to the parasite. However, irradiated *T. annulata *transformed cells have been shown to stimulate strong lymphocyte proliferation in autologous PBMC from Theileria-naïve animals [45]. Although the nature of the responding cells has not been defined in detail, it can include γδ T cells [46] NK cells and non-MHC-restricted T cells [47], all of which can express CD8. A concern at the outset of the study was that such cells might overgrow the specific T cells directed at the virally expressed antigens. This proved not to be the case. A majority of CD8 T cell clones generated with canarypox-Tp1 and BHV-1 were specific for the respective virally expressed antigens. However, 6 out of 48 Tp1 specific CD8^+ ^clones with detectable cytolytic activity killed both antigen-pulsed and unpulsed Theileria-infected targets. The precise lineage of these cells was not determined, but given the previous evidence of cellular responses of naïve PBMC to *T. annulata*, they probably represented an innate response to the APC.

In conclusion, the current study has demonstrated that *T. annulata *transformed monocytes can be used as APC to stimulate and maintain cultures of virus-specific CD8 T cell lines in vitro. Such T cell lines will be of particular value for identification of antigens and epitopes recognised by responding T cells and for determining the composition of antigenic specificities within CD8 T cell responses. In the case of BHV-1, the ability of the virus to infect *T. annulata*-transformed cells facilitates analyses of responses to the whole virus. For other viruses that do not infect these cells, infection with recombinant poxviruses can be used to analyse responses to individual viral proteins. The ease with which *T. annulata*-transformed cell lines can be established and maintained in vitro offers a number of practical advantages over other autologous APC such as monocytes and dendritic cells. There is no limitation in the numbers of available cells, they do not require repeated bleeding of animals or lengthy isolation procedures and they can be used to analyse responses in small aliquots of cryopreserved PBMC from cattle that are no longer alive.

## Abbreviations

BHV-1: bovine herpesvirus 1; PBMC: peripheral blood mononuclear cells; APC: antigen presenting cells; MHC: major histocompatibility complex; gB, gC, gD: glycoproteins B, C and D; TCID_50 _: tissue culture infectious dose 50; Gy: Gray; CFSE: carboxyl-fluorescein succinyl ester; IFN-γ: interferon gamma; IL-2: interleukin 2; NK cell: natural killer cell.

## Competing interests

The authors declare that they have no financial, personal or professional interests that would have influenced the content of the paper or interfered with their objective assessment of the manuscript.

## Authors' contributions

JH and NDM carried out the work and JH drafted the manuscript. WIM conceived of the study, and participated in its design and coordination and helped to draft the manuscript. All authors read and approved the final manuscript.
